# The Association of Life Stress with Subsequent Brain and Cognitive Reserve in Middle-Aged Women

**DOI:** 10.3233/JAD-220923

**Published:** 2023-05-02

**Authors:** Isabel K. Schuurmans, Sanne J.W. Hoepel, Charlotte A.M. Cecil, Manon H.J. Hillegers, M. Arfan Ikram, Annemarie I. Luik

**Affiliations:** a Department of Epidemiology, Erasmus MC University Medical Center Rotterdam, Rotterdam, the Netherlands; b The Generation R Study Group, Erasmus MC University Medical Center Rotterdam, Rotterdam, the Netherlands; c Department of Child and Adolescent Psychiatry and Psychology, Erasmus MC University Medical Center Rotterdam, Rotterdam, the Netherlands; d Department of Biomedical Data Sciences, Molecular Epidemiology, Leiden University Medical Center, Leiden, the Netherlands

**Keywords:** Cognitive reserve, epidemiology, middle aged, psychological, stress

## Abstract

**Background:**

Cognitive and brain reserve refer to individual differences that allow some people to better withstand brain pathology than others. Although early life stress has been recognized as a risk factor for low reserve in late life, no research yet has studied this across midlife.

**Objective:**

To examine the associations of life stress with brain and cognitive reserve in midlife.

**Methods:**

We included 1,232 middle-aged women who participated in the ORACLE Study between 2002-2006). Life stress was calculated as the shared variance of four cumulative stress domains, created from items measured between pregnancy and 10 years after childbirth. Brain reserve was defined as healthy-appearing brain volume measured with MRI; cognitive reserve as better cognitive functioning than expected based on age, education, and brain MRI measures, using structural equation modelling.

**Results:**

More life stress was associated with lower brain (standardized adjusted difference: -0.18 [95% CI 0.25,-0.12]) and cognitive reserve (-0.19 [-0.28,-0.10]). Although, effect sizes were typically smaller, cumulative stress domains were also associated with brain reserve (life events: -0.10 [-0.16,-0.04]; contextual stress: -0.13 [-0.19,-0.07]; parenting-related stress: -0.13[-0.19,-0.07]; interpersonal stress: -0.10 [-0.16,-0.04]) and cognitive reserve (life events: -0.18 [-0.25,-0.11]; contextual stress: -0.15 [-0.10,-0.02]; parenting-related stress: -0.10 [-0.18,-0.03]; interpersonal stress not significant).

**Conclusion:**

Women who experience more life stress in midlife were found to have lower reserve. Effects were primarily driven by shared variance across cumulative stress domains, suggesting that focusing on single domains may underestimate effects. The effect of life stress on lower reserve may make women with stress more prone to neurodegenerative disease later in life than women without stress.

## INTRODUCTION

Neurodegenerative disorders such as Alzheimer’s disease are increasingly recognized as a major health burden, with the number of cases expected to double every 20 years [1]. This has created a pressing need to identify what is underlying these disorders. Neurodegenerative disorders are typically characterized by clinical symptoms such as cognitive decline [2]. Yet, some people show fewer of these clinical symptoms despite having the same amount of brain pathology [3]. This differential susceptibility for brain pathology can be explained by brain reserve and cognitive reserve [4]. Whereas brain reserve is a passive form of reserve that reflects the neurobiological capital available at that time, cognitive reserve is an active form of reserve that reflects adaptability of cognitive processes [5]. The protective effects of reserve are almost exclusively seen later in life but reserve itself is built up already earlier in life. Several factors have been proposed to positively influence reserve, such as high childhood school grades [6], occupational complexity [7], and healthy lifestyle [8]. Conversely, factors such as smoking, diabetes mellitus, depression, and early life stress have been associated with lower reserve in late life [9–11].

Early life stress refers to an individual’s exposure to single or multiple adverse events in prenatal life and childhood [12]. Yet, exposure to adverse events continues beyond childhood. This ‘life stress’ can be defined as exposure to sudden events, chronic demands, or traumas that require an individual to extensively readjust their life [13], for example death or sickness of a loved one and financial strains. Life stress may particularly occur in midlife, a period characterized by several changes and adaptions [14]. For many, midlife is also the period of child-rearing [15]. Child-rearing is generally associated with favorable cognitive outcomes later in life [16], yet, raising a child may also be accompanied by specific stressors [17]. The presence of young and dependent children may for example lead to stress because of the daily demands of parenting, work-family conflict, and parental conflict. While life stress is known to associate to poor mental and physical health outcomes in midlife and beyond [18, 19], the association between life stress and reserve in midlife remains unclear.

In this prospective population-based study, we measured life stress based on 10 years of data in middle-aged women, where the term life stress in this manuscript refers to the exposure of events, not the response to the events. Using structural equation modelling, we first examined the associations of a latent factor of life stress with both brain reserve and cognitive reserve. Second, we examined the association between cumulative stress domains and reserve over and above other cumulative stress domains, to establish whether associations were driven by specific stress domains. We expected that more life stress would be associated with less reserve. No initial hypotheses were specified for which of the cumulative stress domains would be driving the associations. Further, sensitivity analyses were used to additionally examine associations in a subgroup of women without clinically relevant depressive symptoms and across country of origin (non-Western versus Western).

## MATERIALS AND METHODS

### Population

The current study included women participating in the Origins of Alzheimer’s Disease Across the Life course (ORACLE) Study, which focusses on the life-course study of brain health [20]. The ORACLE Study is embedded in The Generation R Study, a population-based prospective cohort from fetal life onwards [21]. In short, pregnant mothers that were residents of the study area (Rotterdam, the Netherlands) and had a delivery date between April 2002 and January 2006 were eligible to enroll. In total, 9,778 mothers with partners and children were enrolled. Approximately 15 years after initial inclusion, 1,362 mothers and 721 partners were invited to take part in the ORACLE Study, also including brain magnetic resonance imaging (MRI) and cognitive testing [20]. A timeline of study assessments is shown in Supplementary Figure 1.

The current study included only women participating in the ORACLE Study (*N* = 1,362); men did not have enough data available to participate as well. We excluded women missing more than 50% of the life stress items (*N* = 18), without T1-weighted images (*N* = 47), with incidental findings (i.e., brain pathologies that can bias brain structure estimates, for instance meningioma > 3 cm) (*N* = 7), with T1-weighted images of insufficient quality (*N* = 28), missing more than three out of six cognitive tests (*N* = 25), or without information on education (*N* = 5). The final study sample included 1,232 participants. This study included only women due to limited data available in partners.

General design, research aims, and specific measurements of The Generation R Study have been approved by the Medical Ethical Committee of the Erasmus Medical Center, in accordance with the Declaration of Helsinki of the World Medical Association. Written informed consent was obtained from all participants.

### Life stress

**Fig. 1:**
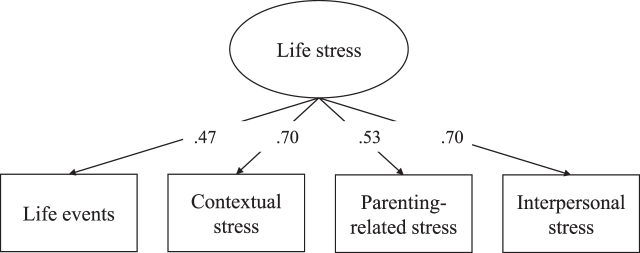
Factor loadings life stress measure.

A life stress measure was created using items measured from pregnancy onwards up to 10 years after childbirth (Hoepel et al., unpublished data), consistent with earlier work in The Generation R Study [22]. Stressors were derived from self-report questionnaires. Multiple stressors were measured repeatedly over time. The repeated time points were combined, and a stressor was coded as ‘1 = *present*’ whenever a stressor was present at any of the available time points, and as ‘0 = *not present*’ whenever a stressor was absent at all of the time points. On top of that, the stressor ‘low income’ was *additionally* combined into a chronic item; the stressor ‘unemployment’ was *instead* combined into a chronic item. Chronic items were coded as ‘1 = *present*’ whenever the stressor was present on all time points, and as ‘0 = *not present*’ if the stressor was absent at any of the three time points. See Supplementary Table 1 for an overview of the stressors. A total of 42 single stressors were summed into four cumulative stress domains: i) *life events* (e.g., sickness in family and friends, history of childhood abuse), ii) *contextual stress* (e.g., unemployment, study stress, iii) *parenting stress* (e.g., lack of maternal confidence, health of child), and iv) *interpersonal stress* (e.g., divorce, family distress); note, each cumulative stress domain summed a different set of stressors. Life stress was estimated as a reflective latent variable using the four cumulative stress domains as indicators measuring what is common (i.e., shared) between different cumulative stress domains, with higher scores implying that more life stress is present. The model had a good fit: chi-square statistics (*χ*^2^) (2)=0.81, *p* = 0.666, standardized root mean square residual (SRMR)=0.01, and comparative fit index (CFI)=1.00 (factor loadings shown in Fig. 1). In contrast to previous work (Hoepel et al., unpublished data), education was not included in our life stress measure as it is part of our outcome.

### Brain reserve

Brain reserve encompasses all the anatomical or structural aspects of the brain but is exclusive of neuropathology [23]. Therefore, we defined brain reserve as healthy-appearing brain volume, which was calculated as total brain volume minus white matter lesion volume (cm^3^) such that brain reserve denotes the neural capacity to buffer neuropathology, following previous literature [24, 25]. Total brain volume (sum of cerebral white matter and total gray matter) and white matter lesion volume were measured using T1-weighted images. The images were acquired during follow-up, 15 years post childbirth (*M* = 14.6, *SD* = 0.9). The imaging protocol and scanning parameters have been described elsewhere [20]. In short, T-weighted images were acquired using the 3 Tesla GE Discovery MR750 w MRI (General Electric, Milwaukee, WI, USA) with an 8-channel head coil. Images were processed using FreeSurfer image analysis suite 6.0 (http://surfer.nmr.mgh.harvard.edu), and were segmented into prespecified cortical and subcortical regions, brain volume and white matter lesions.

### Cognitive reserve

Cognitive reserve was also measured at follow-up, and was calculated using six different cognitive tests, demographic information, and structural brain MRI measures. Cognitive tests are extensively described elsewhere [20]. In short, cognitive tests were administered in Dutch, and only in participants that were sufficient Dutch speakers. We administered six tests, being the 15-word learning test was administered to assess verbal learning and verbal memory [26]; the Stroop task to assess selective attention and automaticity [27]; the letter-digit substitution test to assess processing speed and executive function [28]; the word fluency test to measure efficiency of searching the long term memory [29]; the Purdue pegboard test to assess dexterity and fine motor skills [30]; and the design organization test to measure visuospatial ability [31]. As the Purdue pegboard test and the design organization test were introduced into the test battery later into the study, these were only administered in 66.8% and 79.2% of the participants, respectively. Demographic information included age during cognitive tests and education level. Education level was self-reported at intake [21]. When education level was not available at intake, we used education level reported at 3 years post childbirth (*N* = 12, 1.0%) or 5 years post childbirth (*N* = 21, 1.7%). Education was categorized according to the classification of Statistics Netherlands [32], separating low education (up to 3 years or less at secondary education or completed pre-vocational education), middle (more than 3 years of secondary education or completed vocational education), and higher education (completed higher professional education or university). Structural brain MRI measures included total brain volume and white matter lesion volume. The distribution of white matter lesion volume was skewed; hence the variable was log-transformed.

**Fig. 2:**
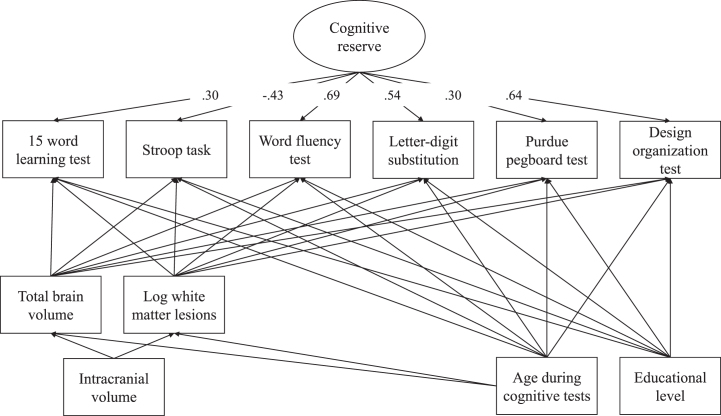
Factor loadings cognitive reserve model.

Cognitive reserve was quantified using the residual approach [24, 33], such that a higher cognitive reserve reflects better cognitive functioning than expected based on demographics and structural brain MRI measures, following previous literature [9, 24, 25]. Using structural equation modelling, cognitive reserve was calculated as a reflective latent variable using six different cognitive tests as indicators. The score for each cognitive test was adjusted for sociodemographic variables (i.e., age and education status) and structural brain MRI measures (i.e., total brain volume and log-transformed white matter lesion volume). Structural brain MRI measures were additionally adjusted for intracranial volume and age. The model had a good fit: *χ*^2^ (20)=88.56, *p* < 0.001, SRMR = 0.02, and CFI = 0.98 (factor loadings shown in Fig. 2).

### Other variables

Depressive symptomatology was assessed at follow-up with the Dutch version of the Center for Epidemiologic Studies Depression Scale (CES-D), which is a self-report questionnaire developed to measure depressive symptomatology among the general population [34, 35]. A score of 16 of higher (range 0-60) was defined as clinically relevant depressive symptoms [35]. Country of origin was categorized according to the classification of Statistics Netherlands [36], which distinguishes ‘Western’ (European, North-American, and Oceanian) and ‘non-Western’ (Central/South American, Asian (excl. Japan), African; see for a more detailed overview Supplementary Table 2).

### Statistical analyses

Missing value frequencies ranged between 0.1-32.4% (*M* = 7.2, SD = 8.5) for stressors and between 0.1-33.0% (*M* = 9.4, SD = 14.1) for cognitive tests. We imputed missing information with Multivariate Imputation by Chained Equations (mice) [37], using a strategy based on Van Buuren [38]. Missing stressors, cognitive tests, and covariates were imputed using the following information: 1) items specific to the cumulative stress domain (stressors only), 2) cumulative stress domain sum scores, 3) other cognitive tests (stressors were imputed using only the letter-digit substitution test), and 4) auxiliary variables (i.e., parity, smoking during pregnancy, age of the mother at intake, BMI at intake, having immigrated, low education level, ever divorced, and family distress). Cumulative stress domain scores were passively imputed, meaning that for each completed imputed dataset, cumulative stress domain scores were computed by summing its stressors and dividing this by the total number of stressors within that domain. We constructed 30 imputed datasets and 60 iterations. All analyses were performed using this imputed data. Pooled estimates were obtained using Rubin’s rules [39].

The primary aim of the study was to examine associations between life stress and reserve, using separate models for brain reserve and cognitive reserve, see Supplementary Figures 2 and 3. For secondary aims we assessed the association of each individual cumulative stress domain with reserve in singular (i.e., linear regression model with one cumulative stress domain as predictor and reserve as outcome) and mutually adjusted models (i.e., linear regression with all four cumulative stress domains as predictors and reserve as outcome). We used sensitivity analyses to assess the associations in a subgroup of women without clinically relevant depressive symptoms and across country of origin (non-Western versus Western).

All analyses were performed with R version 3.6.3 [40]. Associations were examined using structural equation modeling within the Lavaan package version 0.6–9 [41]. Two separate structural equation models were created; a first model investigating associations of life stress with brain reserve and a second model investigating associations of life stress with cognitive reserve. Beta coefficients from all models are interpreted as standardized adjusted differences, with beta coefficients representing the standardized adjusted mean difference in the outcome (expressed as z-score) per 1-SD increase in life stress.

## RESULTS

### Descriptive statistics

The sample consisted of women with a mean age of 46.6 (SD = 4.5) during the cognitive assessment. Compared to the excluded sample, the included sample had a more favorable socioeconomic status, had less prenatal depressive symptoms, and their children had an older gestational age and higher birth weight (Supplementary Table 3). Most of the included sample had received middle (55.4%) or high (32.4%) education (Table 1). A total of 288 participants (23.4%) had a non-Western country of origin, particularly Suriname (7.1%), Indonesia (3.7%), and Turkey (3.0%) (Supplementary Table 2). A correlation matrix for all variables included in analyses can be found in Supplementary Table 4. As cognitive reserve is corrected for structural brain MRI measures, which strongly overlap with brain reserve, we find low correlation between brain and cognitive reserve (*r* = 0.00, *p* = 0.997).

**Table 1:** Sociodemographic characteristics study sample

	M (SD)	*N* (%)
*N*		1,232
Age at follow up, y	46.6 (4.5)	
Education level		
Low		151 (12.3)
Middle		682 (55.4)
High		399 (32.4)
Ethnicity		
Non-Western		288 (23.4)
Western		943 (76.5)
Marital status		
No partner		96 (7.8)
Partner		1,099 (89.2)
Household income		
Low (<€1200 monthly)		96 (7.8)
Medium to high		970 (78.7)
Clinically relevant depressive symptoms		139 (11.3)

Education level was measured during intake, and if not available 3 to 5 years post childbirth. Ethnicity, marital status, and household income were measured during intake. Depressive symptoms were measured 15 years post childbirth. Missing information ranged between 0.1 and 11.3%.

### Life stress and reserve

Life stress was associated with lower brain reserve (standardized adjusted difference: -0.18 [95% CI -0.25,-0.12]) and lower cognitive reserve (standardized adjusted difference: -0.19 [-0.28,-0.10]) at follow-up. Both models had an acceptable fit (brain reserve: *χ*^2^ (9)=75.77, *p* < 0.001, SRMR = 0.06, and CFI = 0.92; cognitive reserve: brain reserve: *χ*^2^ (69)=332.30, *p* < 0.001, SRMR = 0.06, and CFI = 0.93). Secondary analyses showed that in singular models all cumulative stress domains were individually associated to lower brain reserve (standardized adjusted difference; life events: -0.10 [-0.16,-0.04]; contextual stress: -0.13 [-0.19,-0.07]; parenting-related stress: -0.13 [-0.19,-0.07]; interpersonal stress: -0.10 [-0.16,-0.04]), and all cumulative stress domains except for interpersonal stress were individually associated with lower cognitive reserve (standardized adjusted difference; life events: -0.18 [-0.25,-0.11]; contextual stress: -0.15 [-0.10,-0.02]; parenting-related stress: -0.10 [-0.18,-0.03]), see Table 2. In a mutually adjusted model including all cumulative stress domains, only contextual stress and parenting-related stress remained associated to brain reserve (standardized adjusted difference: contextual stress: -0.08 [-0.14,-0.01]); parenting-related stress: -0.08 [-0.15,-0.02]), while only life events and contextual stress remained significantly associated to cognitive reserve (standardized adjusted difference; life events: -0.16 [-0.23,-0.09]; contextual stress: -0.10 [-0.20,-0.01]), see Supplementary Table 5.

**Table 2:** The association of life stress with brain reserve and cognitive reserve

	Brain reserve			Cognitive reserve		
	Adj. dif.	95% CI	*p*	Adj. dif.	95% CI	*p*
Life stress	–0.18	–0.25, –0.12	<0.001	–0.19	–0.28, –0.10	<0.001
Life events	–0.10	–0.16, –0.04	0.001	–0.18	–0.25, –0.11	<0.001
Contextual stress	–0.13	–0.19, –0.07	<0.001	–0.15	–0.22, –0.07	<0.0001
Parenting-related stress	–0.13	–0.19, –0.07	<0.001	–0.10	–0.18, –0.03	0.005
Interpersonal stress	–0.10	–0.16, –0.04	0.001	–0.06	–0.13, 0.01	0.106

Adj. dif., standardized adjusted difference in the outcome per 1-SD increase in stress. Each row corresponds to one model output.

### Sensitivity analyses

When analyses were restricted to women without clinically relevant depressive symptoms, associations between life stress and brain reserve slightly attenuated (standardized adjusted difference: -0.14 [-0.23,-0.06]) and associations with cognitive reserve slightly strengthened (standardized adjusted difference: -0.22 [-0.33,-0.11]). Associations between the cumulative stress domains and reserve also only slightly attenuated, see Supplementary Table 6.

When analyses were stratified for country of origin, the association of life stress showed smaller effect sizes for brain reserve (standardized adjusted difference; non-Western: -0.12 [-0.27,0.03]; Western: -0.11 [-0.18,-0.03]) and cognitive reserve (standardized adjusted difference; non-Western: -0.10 [-0.29,0.08]; Western: -0.08 [-0.18,0.03]). Associations between the cumulative stress domains and reserve also attenuated, see Supplementary Tables 7 and 8, with a clear exemption for parenting-related stress, that showed stronger effect sizes in non-Western women than in Western women for both brain reserve (standardized adjusted difference: -0.17 [-0.31,-0.03]) and cognitive reserve (standardized adjusted difference: -0.15 [-0.29,-0.01]). Of note, non-Western women reported higher levels of parenting-related stress than Western women (standardized difference: 0.45 [0.31, 0.58]).

## DISCUSSION

In this population-based sample of middle-aged women, we found that life stress measured over the course of 10 years was associated with lower brain reserve and cognitive reserve during midlife, in accordance with our hypothesis. Overall life stress was more strongly associated with reserve than individual cumulative stress domains. Findings were largely consistent when restricting analyses to women without clinically relevant depressive symptoms. When analyses were stratified for country of origin, results attenuated in both groups, although we identified strong associations between the parenting-related stress domain and reserve for non-Western women. Together, these findings point to stress during midlife as a potential risk factor for lower cognitive and brain reserve, indicating that not only stress early in life is associated with lower reserve [10, 11].

Our findings suggest comparably strong associations between life stress and the two types of reserve. Yet, brain reserve and cognitive reserve are two different concepts [5], with potential differences in underlying mechanisms. On the one hand, animal-based research suggests that stress is linked to neuronal capacity, which is the foundation of brain reserve, through pathways including increased release of glucocorticoids as well as through changes in gene expression [11, 42]. On the other hand, stress may be linked to cognitive reserve among others by changes in dopamine transmission, which affects the development and maintenance of the brain network supporting cognitive reserve [43]. Future studies in humans are needed to understand what mechanisms link stress to brain versus cognitive reserve, which can inform intervention and prevention research, such that both brain reserve and cognitive reserve can be targeted in order to decrease the risk of developing neurodegenerative disease [44].

Overall life stress, measured as the shared variance across all stressors, was more strongly associated with reserve than any individual cumulative stress domain. This implies no specific cumulative stress domain is driving associations per se. Earlier work has shown that different domains of stress tend to co-occur [45–47] and that the shared variance between cumulative stress domains is particularly associated with negative outcomes [22, 48, 49]. This could either imply that the co-occurrence of stress is associated with low reserve but may also indicate that risk factors induce each other, suggesting that this accumulation is driving associations. Focusing on single stress domains may therefore underestimate associations with reserve. This also confirms the need to develop comprehensive assessment tools to identify population at risk.

When analyses were stratified for country of origin, effect sizes were typically smaller and associations were not any longer statistically significant. This finding should be interpreted cautiously, given that the majority of participants with a non-Western country of origin may not be tested in their native language. We suggest that this attenuation of effects may be explained by a loss of variability, rather than by decreased sample sizes due to stratification, as confidence intervals did not considerably widen. This loss of variability may imply an important role for country of origin in explaining associations between life stress and reserve, either as confounder or as moderator. This may emerge as women with a non-Western country of origin typically experience more life stress. Of note, 61.1% of the non-Western participants migrated to the Netherlands during their life, which might be a cause of stress in itself. Immigrant families have been found to report higher levels of daily distress and marital discord [50]. On the one hand, this may be due to the process of acculturation [51], which is accompanied by several potential stressful life changes, such as taking on a minority status, learning a new language, and creating new social support networks. On the other hand, it could also be that non-Western women report more stressors due to cultural differences in answering questions, for instance in the norm governing the experience of emotions [52]. We found that particularly parenting-related stress was a strong factor in driving associations for non-Western women. Accordingly, non-Western women reported more parenting-related stressors in our study. This was consistent with an earlier study in our research group that showed that mothers with more unfavorable immigration reported higher levels of child behavioral problems [53], which may complicate parenting and heighten stress. Potentially, stress prevention may benefit from more culture-sensitive approaches, particularly identifying people with an immigration background as population-at-risk.

In this population-based study, we measured life stress using a wide range of stressors and calculated brain reserve and cognitive reserve using several indicators inferred from cognitive assessment and MRI. Yet, our work should be interpreted in light of the following limitations. First, the study included only women due to limited data available in partners. Therefore, it is unclear to what extent results generalize to men. Second, we excluded participants with high frequencies of missing data. Although we undertook efforts to exclude as few participants as possible by imputing predictors and outcomes, our results may be influenced by selection bias, as increased stress is associated with higher rates of missing data [54]. Third, the life stress measure was built from cumulative stress domains. Therefore, no information on the relative strength or duration of stressors was taken into account. Only a few stressors were measured repeated to take into account the chronic nature of the exposure into account. Also, this study did primarily include stressors reflecting potentially stressful events (e.g., death or sickness of a loved one and financial strains), thus we cannot draw any conclusions about the internal perceptions of stress. Future research should cross-check our outcomes with perceived stress. Finally, caution should be taken in the interpretation of cognitive reserve, which was estimated using a residual-based method. Previous literature raised the concern that with this method, cognitive reserve may mainly capture cognitive performance [55]. Therefore, our conclusions may only apply to cognitive *performance*, and in a lesser extent to cognitive reserve. We observed that correlations between cognitive reserve and cognitive performance were moderate to large. This may indicate that the residual in this study indeed reflects a cognitive reserve and goes beyond current cognitive performance alone, but at least in part it could also reflect cognitive performance, controlled for age, education, and brain factors per se.

In conclusion, women who experienced more stress were found to have lower reserve already in middle-age, which might put them at risk for the development of neurodegenerative disease later in life [44]. Effects were primarily driven by shared variance across different cumulative stress domains, suggesting focusing on single domains of stress may underestimate effects. Country of origin came forward as a potential important confounder, explaining associations between stress and reserve. Together, these findings point to stress during the life course as a potential target for enhancing reserve.

## Supplementary Material

Supplementary Material

## Data Availability

Because of restrictions based on privacy regulations and informed consent of the participants, data cannot be made freely available in a public repository. However, data can be obtained upon request (datamanagementgenr@erasmusmc.nl).
