# Supplementary material for: The Association of Life Stress with Subsequent Brain and Cognitive Reserve in Middle-Aged Women
Source: J Alzheimers Dis. 2023 May 2;93(1):97–106. doi: 10.3233/JAD-220923 (PMC10200172; doi:10.3233/JAD-220923)
Supplement: Supplementary Material [file jad-93-jad220923-s001.pdf]

Supplementary Material

The Association of Life Stress with Subsequent Brain and Cognitive Reserve in Middle-Aged Women

Supplementary Figure 1. Timeline of study assessments

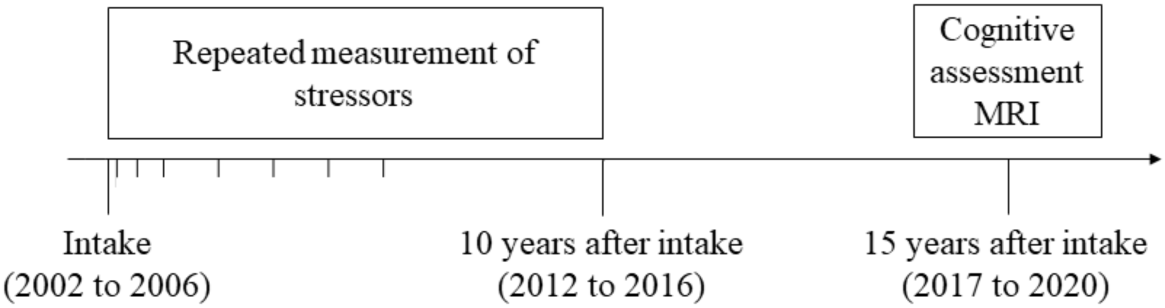

Supplementary Figure 2. Structural equation model association life stress and brain reserve

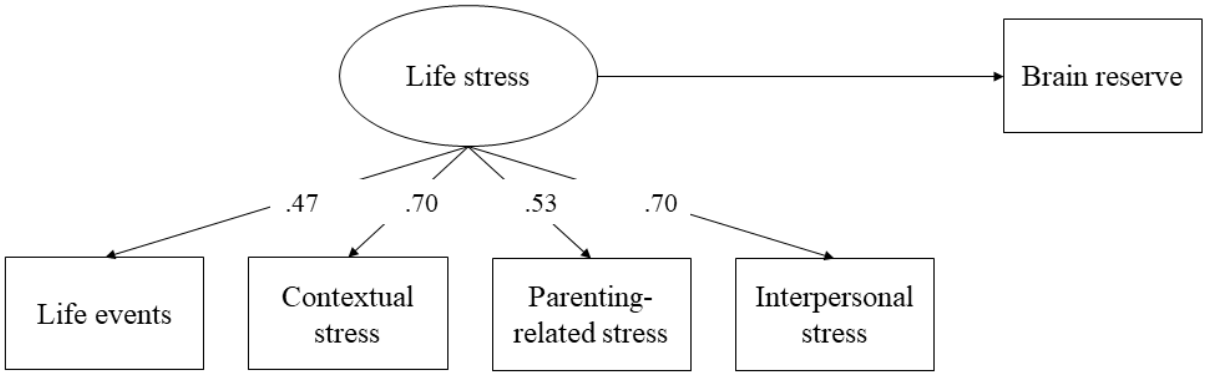

Supplementary Figure 3. Structural equation model association life stress and cognitive reserve

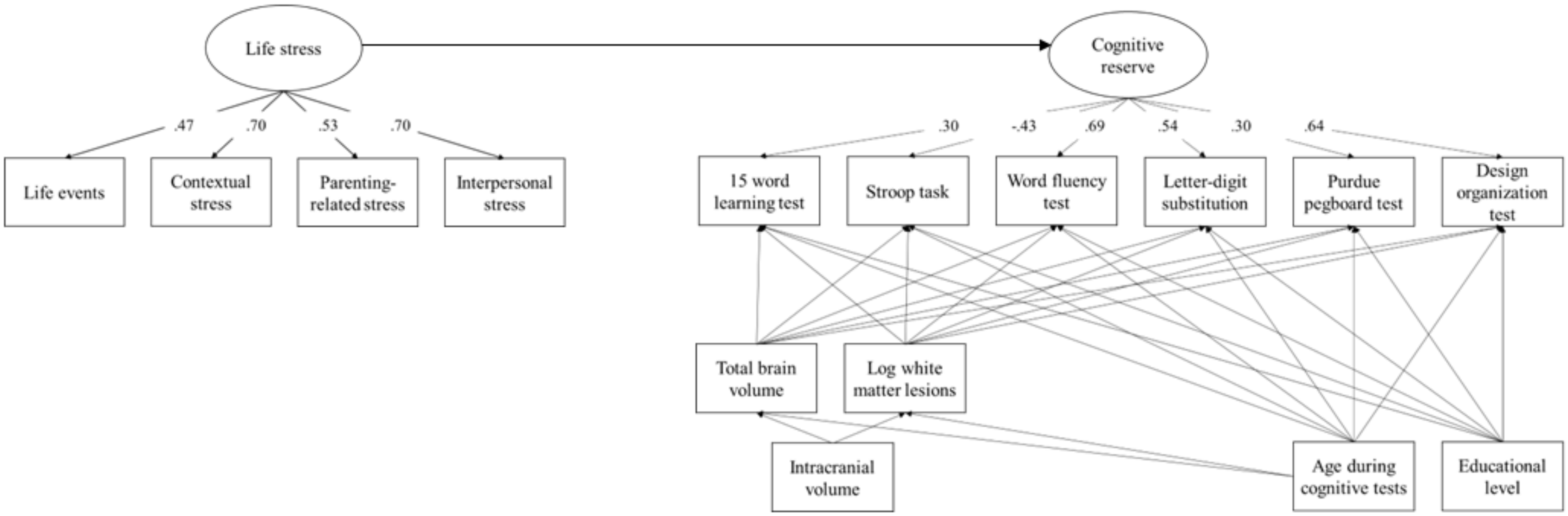

Supplementary Table 1. Overview stressors per cumulative stress domain

LIFE EVENTS	Question; *answer considered as risk*	Time point (after birth)	Reference
1. Death of child or partner	Has one of your children or your partner died? *Yes.*| Is the father/mother or other caretaker still alive? *No.*| What is your marital status? *Widow.*	prenatal, 2 y, 5 y, 9 y	[1-3]
2. Death of someone else	Has your father or mother (in-law) a brother or sister or a good friend died? *Yes.*| Is your mother still alive? Is your father still alive? *No.*| Have the following things occurred: Death of someone in close proximity. *Yes.*	prenatal, 3 y	[2, 3]
3. Sickness in family or friends	Has your partner or other family member or one of your parents (in-law) been seriously ill? *Yes.*| Are you nursing an ill or handicapped family member or friend who does not live in your home? *Yes.*| Have the following things occurred: problems with physical/mental health of people in close proximity. *Yes.*| Did a family member get seriously sick or did he/she have an accident? *Yes.*	prenatal, 3 y, 9 y	[1, 3, 4]
4. Infertility	Is this pregnancy the result of infertility treatment? *Yes.*| Have you had problems becoming pregnant again? *Yes.*	prenatal, 4 y	[2]
5. Abortion	Have you had an abortion? *Yes.*	9 y	[2]
6. Miscarriage/ stillborn	Have you had: a miscarriage (before 16 weeks)? *Yes.*| A stillborn (after 16 weeks)? *Yes (including ectopic pregnancies, molar pregnancies and vanishing twins).*	9 y	[2]
7. Victim of crime	Have you been a victim of robbery, theft, physical abuse or rape? *Yes.*| Has the following occurred: Fire or burglary. *Yes.*	prenatal, 3 y	[3]
8. Perceived discrimination	I have been taunted and insulted due to my ethnic background? *Yes.* | I have been threatened or attacked due to my ethnic background? *Yes.*	prenatal	[5]
9. Material deprivation during childhood mother	Does your family: have sufficient heating in house during cold weather; pay rent or mortgage without problems; have on average one hot meal per day; own or lease a car; own a washing machine; own a refrigerator; own a telephone; have holidays away from home 1 or more weeks per year. *If the family possessed 6/8 (75%) or less.*	prenatal	[6]
10. Childhood abuse	Sexual abuse ≥ 10, physical abuse ≥ 10 and/or emotional abuse ≥ 13.	prenatal	[7, 8]
11. Childhood neglect	CTQ: physical neglect ≥ 16 and/or emotional neglect ≥ 30.	prenatal	[7, 8]
12. Divorce own parents	Are your parents divorced or separated? *Yes.*	prenatal	[2]

13. Immigration	What is the country of your birth? *If not the Netherlands.*	prenatal	[2]
**CONTEXTUAL STRESS**			
1. Chronic unemployment	Have you become unemployed? *Yes.*| Which description most applies to you at this moment? *I am looking for work, I am disabled, I live off a social benefit allowance; I am a housewife; I am a school pupil/student* | Has the following occurred: Unemployment. *Yes.*| Did one of the parents involuntary become unemployed? *Yes. Risk should be present at each time point.*	prenatal, 12 mo, 2 y, 3 y, 5 y, 9 y	[1, 3, 9]
2. Trouble paying	Have you experienced any difficulty in paying for food, rent, electricity bill and suchlike? *Yes.*	prenatal, 2 y, 3 y, 9 y	[2]
3. Financial difficulties	Have you had any financial problems? *Yes.*| Have the following things occurred: financial problem. *Yes.*| Does your family have financial difficulties or did your family ever have them? *Yes.*	prenatal, 3 y, 9 y	[3, 4]
4. Low household income	Indicate the net income of your household (per month). ≤ *10th percentile.*	prenatal, 2 y, 3 y, 5 y, 9 y	[9]
5. Chronic low household income*	"…" *low income at each time point*	All time points	[9]
6. Work or study stress	Have you had problems with or at your work during the past year? *Yes.*| Have you had problems at school or with your studies during the past year? *Yes.*| Have the following things occurred: tension at the parent's work that has been felt at home. *Yes.*	prenatal, 3 y	[3, 4]
7. Material deprivation	Does your family: have sufficient heating in house during cold weather; pay rent or mortgage without problems; have on average one hot meal per day; own or lease a car; own a washing machine; own a refrigerator; own a telephone; have holidays away from home 1 or more weeks per year. *If the family possessed 6/8 (75%) or less.*	Prenatal, 9 y	[6]
8. Neighborhood problems	Have you had problems with the residential environment in your street or neighborhood? *Yes.* |Are/were there problems in the neighborhood? (e.g., vandalism or insecurity) *Yes.* | Have you often had problems with vandalism, grime, crime etc. in your neighborhood? *Yes.*	prenatal, 9 y	[1, 4, 9]
9. Arrested by police	Have you ever been arrested by the police? *Yes.*	prenatal	[2]
**PARENTING-RELATED STRESS**			
1. Parenting stress at 18 months	I have much more trouble raising children than I thought. *Yes* | I often feel that I can't cope with things very well. *Yes.*	18 mo	[10]

2. Parenting stress at 8 years	Being a parent is more difficult than I thought. *Yes* | I feel that I can't cope with raising children very well. *Yes.*	8 y	[10]
3. Unplanned pregnancy	Was this pregnancy planned? *No.*	prenatal	[2]
4. Worried about pregnancy outcome	Pregnancy Outcome Questionnaire. *Weighted sum score > 30.*	prenatal	[11]
5. Direct victimization of the child	Did someone ever use physical violence against your child (e.g., beating him/her up)? *Yes.*| Did somebody almost use physical violence against your child? *Yes.*| Did anybody make sexual comments or movements towards your child? *Yes.*| Has your child experienced inappropriate sexual behavior? *Yes.*	9 y	[1]
6. Bad general health child	How would you describe your child's general health? *Bad or moderate.* | How often is your child ill and does it use healthcare facilities? *Slightly more often, repeatedly, almost always.* | How would you rate you child's health in the past 6 months? *Frequently sick or almost always sick.*	6 mo, 12 mo, 2 y, 3 y, 4 y, 5 y, 9 y	[2, 12, 13]
7. Hospitalization or accident of child (needs to be more than once)	Was your child admitted to the children's ward of a hospital in its first week? *Yes.* | Has your child ever had an accident at or near home or with traffic that needed treatment by a GP, doctor at the emergency help or admittance to the hospital? *Yes.* | How often has your child been admitted to the hospital in the last 6 months? *Once or more*| Has your child been injured as the result of an accident and as a resulted needed a doctor or hospital visit? *Yes.*| How often during the past year has your child gone into hospital? *At least once.* | How often have you taken your child to the doctor/emergency room in the past year? *At least once.* | For each broken bone that your child had, please indicate the extent of the trauma. *Average trauma or severe trauma*. | Did your child get very sick or did he/she get an accident? *Yes.*	2 mo, 6 mo, 12 mo, 2 y, 5 y, 9 y	[1, 2]
8. Problems with child's physical health affect parent	Did you have less time for yourself in the last 4 weeks due to your child's physical health? *Yes.* | Have you been worried in the last 4 weeks due your child's physical health? *Yes.* | I worry more about my child's health than other parents do. *Yes.*	12 mo, 2 y, 4 y, 5 y, 9 y	[12]
9. Total problem behavior	Total problem score of the CBCL. *T-score above 63 at any of the time points.*	18 mo, 3 y, 5 y, 9 y	[14, 15]
10. Callous unemotional traits	Interpersonal Callousness questionnaire. *If 80th percentile or higher.*	9 y	[16]

11. Child's emotional and/or behavioral problems affect parents	Did you have less time for yourself in the last 4 weeks due to your child's emotional well-being or behavior? *Yes.* |Have you been worried in the last 4 weeks due to your child's emotional well-being or behavior? *Yes.*	12 mo; 2 y, 4 y, 5 y	[12]
12. Child has conflicts	Does your child have ongoing conflicts with a family member or did your child ever have such a conflict? *Yes.* | Does your child have long-lasting conflicts with someone outside the family or did they ever have those? *Yes.*	9 y	[1]
13. Child bullies or is victim of bullying	Has your child been bullied or has it bullied other children at least once a week? *Yes.*	8 y	[2]
**INTERPERSONAL STRESS**			
1. No partner	Do you currently have a partner? *No.*| Do you have the same partner as during your Gen R pregnancy? *No, I have no partner.*	prenatal, 6 mo, 2 y, 3 y, 4 y, 5 y	[2]
2. Divorce/separation	Have you had a divorce or broken off the relationship with your partner? *Yes.*| What is your marital status? *Divorced.* | Has the following occurred? Divorce. *Yes.*| Are you and your partner divorced or separated? *Yes.*	prenatal, 2 y, 3 y, 5 y, 9 y	[1-3]
3. Relationship problems	Has the following occurred? Problems with marriage relations. *Yes.*| In the past year, have there been difficulties between you and your partner? *Yes.*	prenatal, 3 y	[3, 4]
4. Family distress	Family distress, > *2.17 at least one of the time points.*	prenatal, 5 y, 9 y	[17]
5. Family conflicts	How would you describe the way the members of your family interact with each other? *Poor or average.*| Do other family members have ongoing conflicts with each other (of did they ever have them)? *Yes.*	12 mo, 2 y, 4 y, 5 y, 9 y	[1, 12]
6. Difficulties with family, friends, or others	In the past year, have there been: difficulties in or with your contact with others? *Yes.*| Difficulties between you and your parents or parents-in-law? *Yes.* |Difficulties between you and one or more brothers/sisters? *Yes.*| Problems with people who are or were your friends? *Yes.* | Problems with one or more people from your neighborhood? *Yes.* | Fighting with neighbors, friends or family? *Yes.*	prenatal, 3 y	[3, 4]
7. Lack of social support	Social Support List, *weighted sum score* $\leq$ *26.*	6 mo	[18]

* correlation item low household income and item chronic low household income is 0.52.

Supplementary Table 2. Overview countries of origin

	N (%)	*N* (%)
Western	943 (76.5)	
Dutch		860 (69.9)
Non-Dutch European		81 (6.6)
North-American		1 (0.1)
Oceanian		1 (0.1)
Non-Western	288 (23.4)	
Surinamese		87 (7.1)
Indonesian		46 (3.7)
Turkish		37 (3.0)
Moroccan		33 (2.7)
Dutch Antillean		21 (2.7)
Cape Verdean		28 (2.3)
Asian (excl. Japan and Indonesia)		18 (1.5)
South/Central American		11 (0.9)
African		7 (0.6)

Supplementary Table 3. Comparison of included sample to non-included sample

	Included<br>*N* (%)	Excluded<br>*N* (%)	*t* (*df*)	χ^2 (*df*)	*p*
N	1,232	130			
Age at follow up, y	46.62 ± 4.45	46.2 ± 5.28	0.86	148.97	0.394
Education level	151 (12.3)	28 (23.7)	11.35	1	0.001
Ethnicity	943 (76.6)	77 (63.6)	9.31	1	0.002
Marital status	1099 (92.0)	96 (86.5)	3.25	1	0.071
Household income	970 (91.0)	75 (81.5)	7.59	1	0.006
Clinically relevant depressive symptoms	870 (86.2)	68 (73.9)	9.18	1	0.002

For continuous outcomes, the included sample was compared to the excluded sample using *t*-tests (presented as *M* ± *SD*). For categorical outcomes, comparison was done using a χ^2-squared test (presented as *N* (%)).

Supplementary Table 4. Correlation matrix

		1	2	3	4	5	6	7	8	9	10	11	12	13	14	15	16	17
1	LE	1***																
2	CS	0.33***	1***															
3	PS	0.23***	0.34***	1***														
4	IS	0.36***	0.48***	0.38***	1***													
5	BR	-0.14***	-0.12***	-0.09*	-0.11**	1***												
6	TBV	-0.13***	-0.12***	-0.09*	-0.11**	1.00***	1***											
7	WML	-0.01	-0.02	0.00	-0.03	0.31***	0.31***	1***										
8	ICV	-0.19***	-0.16***	-0.09*	-0.13***	0.83***	0.83***	0.34***	1***									
9	CR	-0.16***	-0.10**	-0.05	-0.04	0.00	0.00	0.00	0.05	1***								
10	LDST	-0.15***	-0.15***	-0.04	-0.06	0.18***	0.18***	-0.03	0.19***	0.81***	1***							
11	WFT	-0.15***	-0.12***	-0.07	-0.11**	0.17***	0.17***	0.01	0.18***	0.63***	0.46***	1***						
12	WLT	-0.10*	-0.10**	-0.06	-0.03	0.03	0.03	-0.02	0.01	0.35***	0.22***	0.29***	1***					
13	PPB	-0.10*	-0.12**	-0.06	-0.01	0.12***	0.12***	-0.05	.010**	0.35***	0.31***	0.15***	0.11**	1***				
14	STR	0.15***	0.15***	0.09*	0.09**	-0.22***	-0.22***	-0.02	-0.23***	-0.5***	-0.35***	-0.33***	-0.20***	-0.12***	1***			
15	DOT	-0.19***	-0.17***	-0.07	-0.09*	0.28***	0.28***	-0.02	0.30***	0.75***	0.57***	0.43***	0.25***	0.27***	-0.43***	1***		
16	Age	-0.02	-0.23***	-0.07	-0.07*	0.02	0.02	0.20***	0.12***	0.00	-0.08**	0.09**	0.01	-0.08*	-0.04	-0.01	1***	
17	Edu1	0.04	0.06*	0.04	0.05	-0.10***	-0.10***	-0.03	-0.11***	0.00	-0.02	-0.06*	-0.06*	0.03	0.05	-0.06	-0.09***	1***
18	Edu2	-0.12**	-0.24***	-0.09*	-0.16***	0.19***	0.19***	0.04	0.22***	0.00	0.16***	0.20***	0.18***	0.04	-0.19***	0.23***	0.22***	-0.77***

1. LE, life events; 2. CS, contextual stress; 3. PS, parenting-related stress; 4. IS, interpersonal stress; 5. BR, brain reserve; 6. TBV, total brain volume; 7. WML, white matter lesions; 8. ICV, intracranial volume; 9. Age, age at scanning; 10. Edu1, intermediate education, yes; 11. Edu2, high education, yes; 12. LDST, letter digit substitution test; 13. WFT, word fluency test; 14. WLT, 15 word learning test; 15. PPT, Purdue pegboard test; 16. STR, Stroop task; 17. DOT, design organization test. CR, cognitive reserve. *significant at $p < 0.05$, **significant at $p < 0.01$, ***significant at $p < 0.001$.

Supplementary Table 5. The mutually adjusted association of cumulative stress domains with brain reserve and cognitive reserve

	Brain reserve			Cognitive reserve		
	Adj. dif.	95% CI	*p*	Adj. dif.	95% CI	*p*
Life events	-0.05	-0.11, 0.02	0.137	-0.16	-0.23, -0.09	<0.001
Contextual stress	-0.08	-0.14, -0.01	0.033	-0.10	-0.20, -0.01	0.025
Parenting-related stress	-0.08	-0.15, -0.02	0.013	-0.05	-0.13, 0.03	0.207
Interpersonal stress	-0.02	-0.09, 0.05	0.579	0.05	-0.04, 0.14	0.259

Adj. dif., standardized adjusted difference in the outcome per 1-SD increase in stress. Table shows one model output.

Supplementary Table 6. The association of life stress with brain reserve and cognitive reserve – analyses restricted to women without depressive symptoms

	Brain reserve			Cognitive reserve		
	Adj. dif.	95% CI	*p*	Adj. dif.	95% CI	*p*
Singular models						
Life stress	-0.14	-0.23, -0.06	0.001	-0.22	-0.33, -0.11	<0.001
Life events	-0.09	-0.16, -0.02	0.009	-0.20	-0.28, -0.12	<0.001
Contextual stress	-0.09	-0.16, -0.02	0.013	-0.15	-0.24, -0.06	0.002
Parenting-related stress	-0.09	-0.16, -0.02	0.008	-0.14	-0.23, -0.05	0.002
Interpersonal stress	-0.08	-0.15, -0.01	0.018	-0.08	-0.17, 0.00	0.056
Mutually adjusted model						
Life events	-0.06	-0.13, 0.02	0.119	-0.17	-0.25, -0.08	<0.001
Contextual stress	-0.04	-0.12, 0.04	0.374	-0.09	-0.20, 0.02	0.109
Parenting-related stress	-0.06	-0.13, 0.02	0.149	-0.09	-0.18, 0.01	0.087
Interpersonal stress	-0.03	-0.11, 0.05	0.456	0.03	-0.08, 0.13	0.607

Adj. dif., standardized adjusted difference in the outcome per 1-SD increase in stress. For singular models: each row corresponds to one model output. For mutually adjusted model: all four rows correspond to one model output.

Supplementary Table 7. The association of life stress with brain reserve and cognitive reserve – analyses restricted to non-Western women

	Brain reserve			Cognitive reserve		
	Adj. dif.	95% CI	*p*	Adj. dif.	95% CI	*p*
Singular models						
Life stress	-0.12	-0.27, 0.03	0.122	-0.10	-0.29, 0.08	0.267
Life events	-0.09	-0.20, 0.03	0.163	-0.11	-0.23, 0.00	0.060
Contextual stress	-0.05	-0.18, 0.07	0.404	-0.06	-0.18, 0.06	0.308
Parenting-related stress	-0.16	-0.28, -0.04	0.009	-0.14	-0.26, -0.02	0.027
Interpersonal stress	-0.03	-0.15, 0.09	0.617	0.03	-0.11, 0.16	0.696
Mutually adjusted model						
Life events	-0.05	-0.18, 0.08	0.450	-0.10	-0.23, 0.03	0.134
Contextual stress	0.00	-0.15, 0.15	0.999	-0.04	-0.18, 0.11	0.602
Parenting-related stress	-0.17	-0.31, -0.03	0.017	-0.15	-0.29, -0.01	0.030
Interpersonal stress	0.06	-0.09, 0.20	0.437	0.14	-0.02, 0.3	0.086

Adj. dif., standardized adjusted difference in the outcome per 1-SD increase in stress. For singular models: each row corresponds to one model output. For mutually adjusted model: all four rows correspond to one model output.

Supplementary Table 8. The association of life stress with brain reserve and cognitive reserve – analyses restricted to Western women

	Brain reserve			Cognitive reserve		
	Adj. dif.	95% CI	*p*	Adj. dif.	95% CI	*p*
Singular models						
Life stress	-0.11	-0.18, -0.03	0.004	-0.08	-0.18, 0.03	0.144
Life events	-0.01	-0.07, 0.05	0.764	-0.10	-0.19, -0.01	0.029
Contextual stress	-0.08	-0.14, -0.02	0.015	-0.08	-0.18, 0.02	0.133
Parenting-related stress	-0.06	-0.13, 0.00	0.045	-0.01	-0.10, 0.08	0.837
Interpersonal stress	-0.07	-0.13, -0.01	0.026	-0.03	-0.11, 0.06	0.497
Mutually adjusted model						
Life events	0.02	-0.05, 0.09	0.570	-0.09	-0.19, 0.00	0.057
Contextual stress	-0.05	-0.12, 0.02	0.175	-0.07	-0.19, 0.05	0.230
Parenting-related stress	-0.04	-0.11, 0.03	0.254	0.02	-0.08, 0.12	0.689
Interpersonal stress	-0.04	-0.11, 0.03	0.268	0.01	-0.09, 0.12	0.779

Adj. dif., standardized adjusted difference in the outcome per 1-SD increase in stress. For singular models: each row corresponds to one model output. For mutually adjusted model: all four rows correspond to one model output.

REFERENCES

[1] Amone-P'Olak K, Ormel J, Huisman M, Verhulst FC, Oldehinkel AJ, Burger H (2009) Life stressors as mediators of the relation between socioeconomic position and mental health problems in early adolescence: the TRAILS study. *J Am Acad Child Adolesc Psychiatry* **48**, 1031-1038.

[2] Kooijman MN, Kruithof CJ, van Duijn CM, Duijts L, Franco OH, van Ijzendoorn MH, de Jongste JC, Klaver CCW, van der Lugt A, Mackenbach JP (2016) The Generation R Study: design and cohort update 2017. *Eur J Epidemiol* **31**, 1243-1264.

[3] Holmes TH, Rahe RH (1967) The social readjustment rating scale. *J Psychosom Res* **11**, 213-218.

[4] Hendriks AAJ, Ormel J, Van de Willige G (1990) Long lasting difficulties measured with a self-assessment questionnaire and semi structured interview: a theoretical and empirical comparison. *Gedrag Gezond* **18**, 273-283.

[5] Vedder P, van de Vijver FJR (2006) Methodological aspects: Studying adolescents in 13 countries. In *Immigrant Youth In Cultural Transition*, Lawrence Erlbaum, Mahwah, p. 47-70

[6] EU-SILC, Severe material deprivation rate, European Union Statistics on Income and Living Conditions https://ec.europa.eu/eurostat/web/products-datasets/product?code=tespm030, 29/11/2019, Accessed 12/12.

[7] Bernstein DP, Stein JA, Newcomb MD, Walker E, Pogge D, Ahluvalia T, Stokes J, Handelsman L, Medrano M, Desmond D (2003) Development and validation of a brief screening version of the Childhood Trauma Questionnaire. *Child Abuse Neglect* **27**, 169-190.

[8] Rijlaarsdam J, Stevens GWJM, Jansen PW, Ringoot AP, Jaddoe VWV, Hofman A, Ayer L, Verhulst FC, Hudziak JJ, Tiemeier H (2014) Maternal childhood maltreatment and offspring emotional and behavioral problems: Maternal and paternal mechanisms of risk transmission. *Child Maltreat* **19**, 67-78.

[9] Statistics Netherlands, Gezondheidsenquête 1981-1996 en POLS-Gezondheid 1997-2009, https://www.cbs.nl/nl-nl/onze-diensten/methoden/onderzoeksomschrijvingen/korte-onderzoeksbeschrijvingen/gezondheidsenquete-1981-1996-en-pols-gezondheid-1997-2009, Accessed June 2.

[10] De Brock A, Vermulst AA, Gerris JRM, Abidin RR (1992) *Nijmeegse Ouderlijke Stress Index, handleiding experimentele versie [Nijmegen parenting stress index, Experimental version manual],* Pearson Benelux, Lisse, The Netherlands.

[11] Theut SK, Pedersen FA, Zaslow MJ, Rabinovich BA (1988) Pregnancy subsequent to perinatal loss: Parental anxiety and depression. *J Am Acad Child Adolesc Psychiatry* **27**, 289-292.

[12] Raat H, Botterweck AM, Landgraf JM, Hoogeveen WC, Essink-Bot M-L (2005) Reliability and validity of the short form of the child health questionnaire for parents (CHQ-PF28) in large random school based and general population samples. *J Epidemiol Commun H* **59**, 75-82.

[13] Ware Jr JE, Kosinski M, Keller SD (1996) A 12-Item Short-Form Health Survey: construction of scales and preliminary tests of reliability and validity. *Med Care* **34**, 220-233.

[14] Achenbach TM, Rescorla LA (2000) *Manual for the ASEBA preschool forms & profiles,* University of Vermont, Research Center for Children, Youth, and Families, Burlington, VT.

[15] Achenbach TM, Rescorla LA (2001) *Manual for the ASEBA school-age forms & profiles*, University of Vermont, Research Center for Children, Youth, and Families, Burlington, VT.

[16] Pardini D, Obradovic J, Loeber R (2006) Interpersonal callousness, hyperactivity/impulsivity, inattention, and conduct problems as precursors to delinquency persistence in boys: A comparison of three grade-based cohorts. *J Clin Child Adolesc Psychol* **35**, 46-59.

[17] Byles J, Byrne C, Boyle MH, Offord DR (1988) Ontario Child Health Study: reliability and validity of the general functioning subscale of the McMaster Family Assessment Device. *Family Process* **27**, 97-104.

[18] Kempen G, Van Eijk LM (1995) The psychometric properties of the SSL12-I, a short scale for measuring social support in the elderly. *Soc Indic Res* **35**, 303-312.
